# Application of contrast-enhanced ultrasound in the diagnosis of tuberous vas deferens tuberculosis

**DOI:** 10.1186/s12879-023-08886-6

**Published:** 2024-01-02

**Authors:** Wenzhi Zhang, Jie Chu, Jianping Xu, Wei Tang, Gaoyi Yang

**Affiliations:** Department of Ultrasound, Hangzhou Red Cross Hospital (Integrated Chinese and Western Medicine Hospital of Zhejiang Province), Hangzhou, 310003 Zhejiang China

**Keywords:** Contrast-enhanced ultrasound, Vas deferens, Tuberculosis, Male infertility, Reproductive system

## Abstract

**Background:**

To assess the value of contrast-enhanced ultrasound (CEUS) in the diagnosis of tuberous vas deferens tuberculosis (VD TB) and improve the positive diagnostic rate of VD TB.

**Methods:**

CEUS and routine ultrasound (US) images of 17 patients with tuberous VD TB confirmed by surgery, pathology, or laboratory semen examination were retrospectively analyzed and summarized, and the positive rates of both imaging techniques were compared.

**Results:**

The 19 VD lesions of the 17 patients were divided into two types according to the CEUS findings: Type I and Type II, and type II was divided into Types IIa, IIb, and IIc. Of the nodules with transverse diameters > 1 cm, 100% presented as type II. Of the nodules with transverse diameters < 1 cm, 37.5% (3/8) presented as type I and 62.5% (5/8) presented as type II. The sonographic manifestations of tuberous VD TB were hypoechoic and mixed echoic. The positive diagnostic rate was 89.5% for CEUS and 68.4% for US, but the difference was not significant (*χ*^*2*^ = 2.533; *P* = 0.111).

**Conclusions:**

CEUS was able to show the blood supply characteristics of tuberous VD TB, the internal necrosis of nodules was more easily observed by CEUS than by routine US, which is helpful for the diagnosis of tuberous VD TB.

## Introduction

The vas deferens (VD) is a continuation of the epididymis with a thicker wall, a more developed muscular layer, and a small lumen. VD is divided into four segments from top to bottom: the internal inguinal, inguinal, and testicular segments. Vas deferens tuberculosis (VD TB) is the rarest form of tuberculosis of the male genital system, Tuberous VD TB is a common type of TB observed by US. In the course of disease progression, the tuberculosis bacterium further proliferates in the VD wall, which thickens and is further destroyed. Simultaneously, the caseous material blocks the VD lumen, forms local nodules, breaks through the VD wall, and then forms peripheral cold abscesses [1]. Tuberous VD TB is a TB tubercle of the vas deferens that is formed by TB invading the VD. Tuberous VD TB is one of the ultrasound classification types of VD TB, which may be detected in tests for epididymis tuberculosis and prostate tuberculosis. Tuberous VD TB occurs mostly in the spermatic cord, followed by the inguinal region. Tuberous VD TB also may be caused by a blood infection, and it is one of the causes of male infertility. At present, early diagnosis and treatment of VD TB is difficult [2, 3]. Ultrasonography (US) is highly useful in epididymal and testicular tuberculosis, but its use has rarely been reported in VD TB [4–7]. The study aim was to retrospectively evaluate the clinical usefulness of contrast-enhanced US (CEUS) for diagnosis of tuberous VD TB. To the best of our knowledge, this is the largest reported study of tuberous VD TB to date.

## Methods

### Patients

Patients admitted to our hospital from March 2010 to October 2012 who had been examined by US and pathologically diagnosed with tuberous VD TB were retrospectively analyzed and included in this study. This study was approved by the Institutional Review Board of the Hangzhou Red Cross Hospital. Written informed consent was obtained from each patient.

### US and CEUS examinations

A Philips iU22® Ultrasound instrument (Philips, Amsterdam, The Netherlands) with an L12-5 probe used at a frequency of 5.0 to 12.0 MHz, an L9-3 probe used at a frequency of 3.0 to 9.0 MHz, and an L5-1 probe used at a frequency 1.0 to 5.0 MHz was used to perform all imaging examinations. The patient was placed in the supine position with the scrotum and groin fully exposed and was examined first by routine US for continuity and nodules of bilateral VD at the groin, spermatic cord, and testes. We also looked for echogenicity changes in both testicles and epididymis and then used color Doppler flow imaging to detect the state of blood flow to the VD lesions. The contrast agent used was Sonovue® (Bracco Diagnostics Inc., Monroe Township, NJ). Before use, 5 mL of normal saline was diluted and shaken well; then, 4.8 mL was injected through the superficial vein of the elbow, which was followed by rinsing of the flushing tube with 5 mL of normal saline. Pulsed reverse harmonic imaging with a low mechanical index of 0.06 was used in the imaging process. Contrast agent perfusion of the entire VD and its internal nodules was observed by pressing the time key and dynamic storage key when the contrast agent was injected. The images of the whole process were stored on the instrument’s hard disk.

### Statistical analysis

Statistical analysis was performed using SPSS 23.0 statistical software (IBM Statistics for Windows, IBM Corp., Armonk, NY). Counting data of the differences between routine US and CEUS of VD TB were analyzed by performing the χ^2^ test. The tuberous VD TB-positivity rates of CEUS and routine US were compared. Statistical significance was accepted for *P* values of < 0.05.

## Results

### Patients’ clinical characteristics

Seventeen patients with tuberous VD TB confirmed by routine US, six confirmed by surgery, nine confirmed by needle biopsy, and two confirmed by laboratory semen examinations were included in the study. The patients’ ages ranged from 17 to 42 years, with an average of 23.5 ± 3.7 years. The age distribution of the patients was 35 (IQR 31,55) years.The longest course of disease was 2.5 years, and the shortest was 3 months. The course of disease in all patients was 13 (IQR 11,16) months. Generally, all patients were in good condition with stable vital signs. Among the 17 patients, 9 had a history of tuberculosis, 7 had a history of epididymal tuberculosis (3 of these 7 patients had tuberculosis of the stump of the VD after epididymal tuberculectomy), and 4 had complicated TB. The lesion diameter of VD TB was 1.5 (IQR 0.5,2.3) cm in all patients. The descriptions of the condition of the patients are presented in Table 1.

**Table 1:** Characteristics of the included patients

Characteristic		n	%
Patients		17	100
Clinical manifestation	Scrotal mass	5	29.4
	Scrotal pain	2	11.8
	Groin mass	4	23.5
	No clinical symptoms	6	35.3
Unilateral or bilateral disease	Unilateral	15	88.0
	Bilateral	2	11.0
A history of disease	Pulmonary tuberculosis	9	52.9
	Epididymal tuberculosis	7	41.2
	Testis and epididymis tuberculosis	1	5.9
Diagnosis	Pathology	4	23.5
	GeneXpert, microbial culture	2	11.8
	Pathology, GeneXpert, and microbial culture	11	64.7

### US features of tuberous VD TB

Tuberous VD TB is a TB nodule formed in the wall and lumen of the VD after the TB invades the VD. US manifestations are usually local enlargement of the VD, and the enlarged area presents low echogenicity or mixed echogenicity.According to the echo characteristics, tuberous VD TB was classified as the hypoechoic type (12 patients) and mixed echogenicity type (7 patients).

Among the patients with the hypoechoic type, six had tuberous VD TB with calcification. Local enlargement of the VD of the lesion showed elliptical nodules (most with clear boundaries), and the interiors were uniform and hypoechoic. There were different degrees of calcification in the nodules, and intermittent anechoic areas could be seen in the distal VD of the nodules. Color Doppler imaging showed no obvious blood flow signal in the nodules or punctate color blood flow signal around the nodules.

**Fig. 1:**
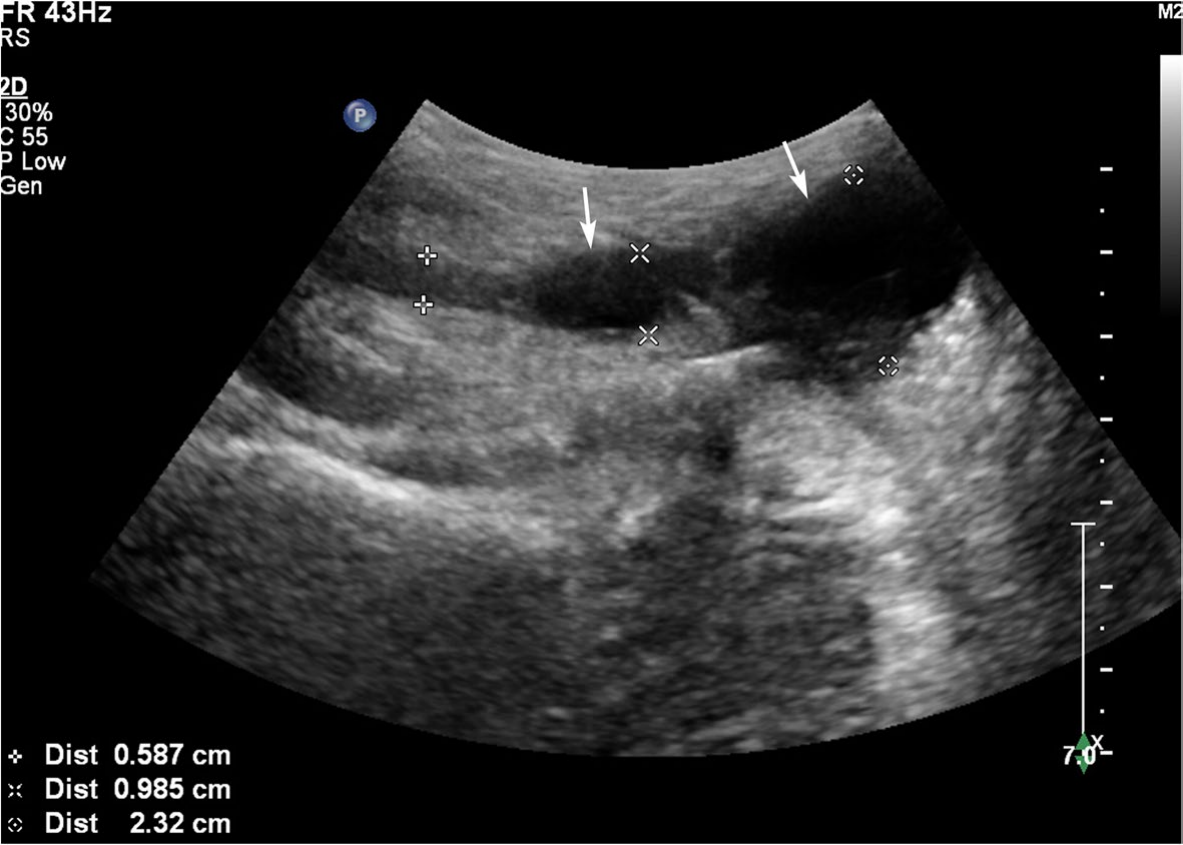
A 31-year-old male with tuberous vas deferens (VD) tuberculosis. The VD wall is thickened with two mixed echo-type nodules showing local enlargement (white arrow)

Among the patients with the mixed echo type (seven patients), US showed thickening of the VD with local nodules. The nodules presented mixed echogenicity with an anechoic area inside, and flocculent sediment echo inside the anechoic area, which could move with the body position (Fig. 1).

### CEUS of tuberous VD TB

**Fig. 2:**
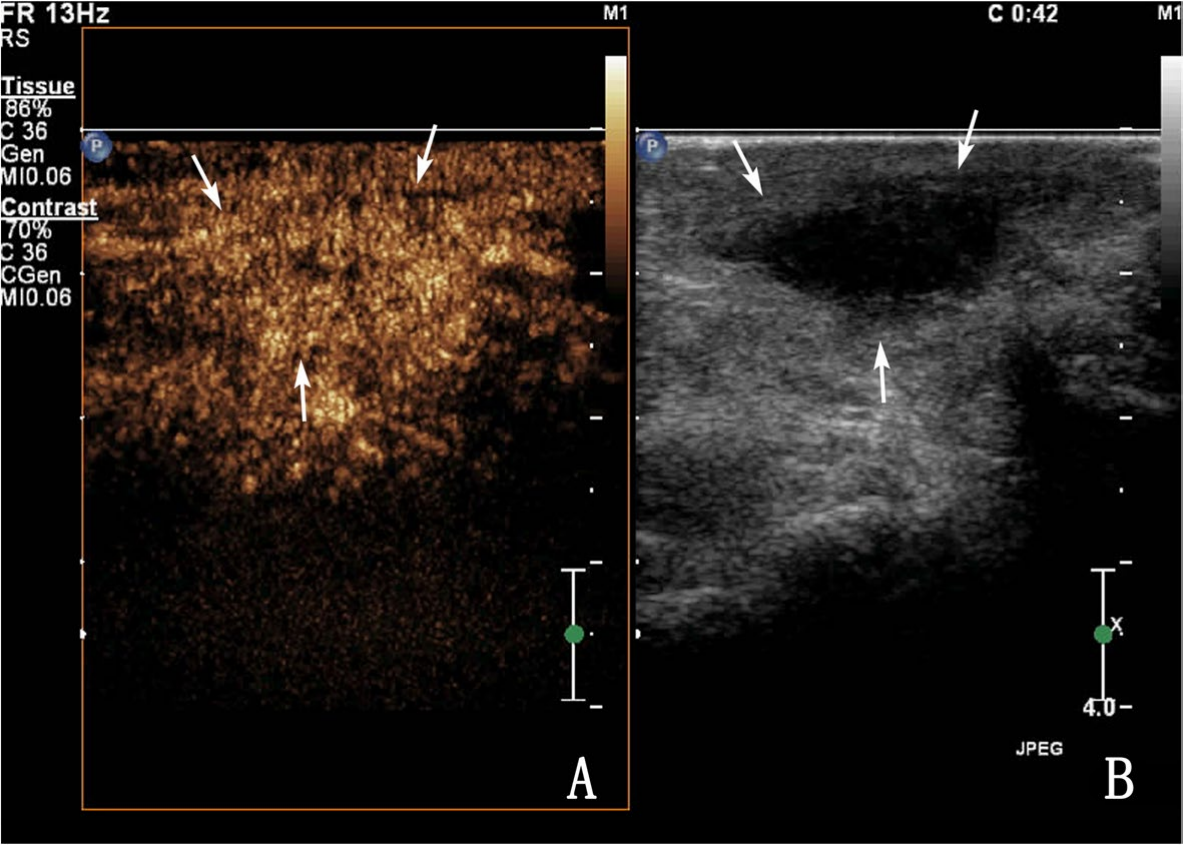
A 43-year-old male with tuberous vas deferens tuberculosis (VD TB). **A** Routine ultrasonography of a VD TB lesion (white arrow). **B** Contrast-enhanced ultrasonography of a VD TB lesion showing homogeneous enhancement (white arrow) (type I)

**Fig. 3:**
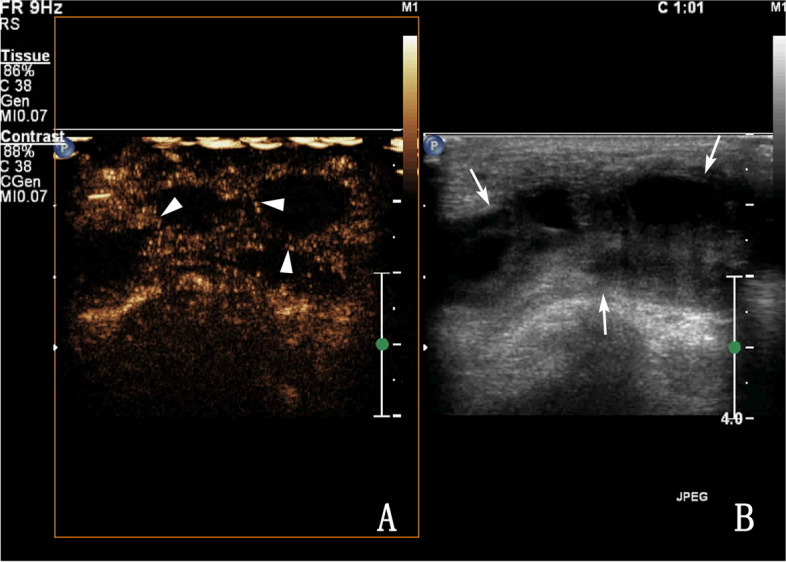
A 33-year-old male with tuberous vas deferens tuberculosis. **A** Routine ultrasonography showing a mixed echo-type lesion (coarse arrow), **B** Contrast-enhanced ultrasonography showing septal enhancement of the lesion (fine arrow), visible inside the irregular areas, not the enhanced area (type IIa)

**Fig. 4:**
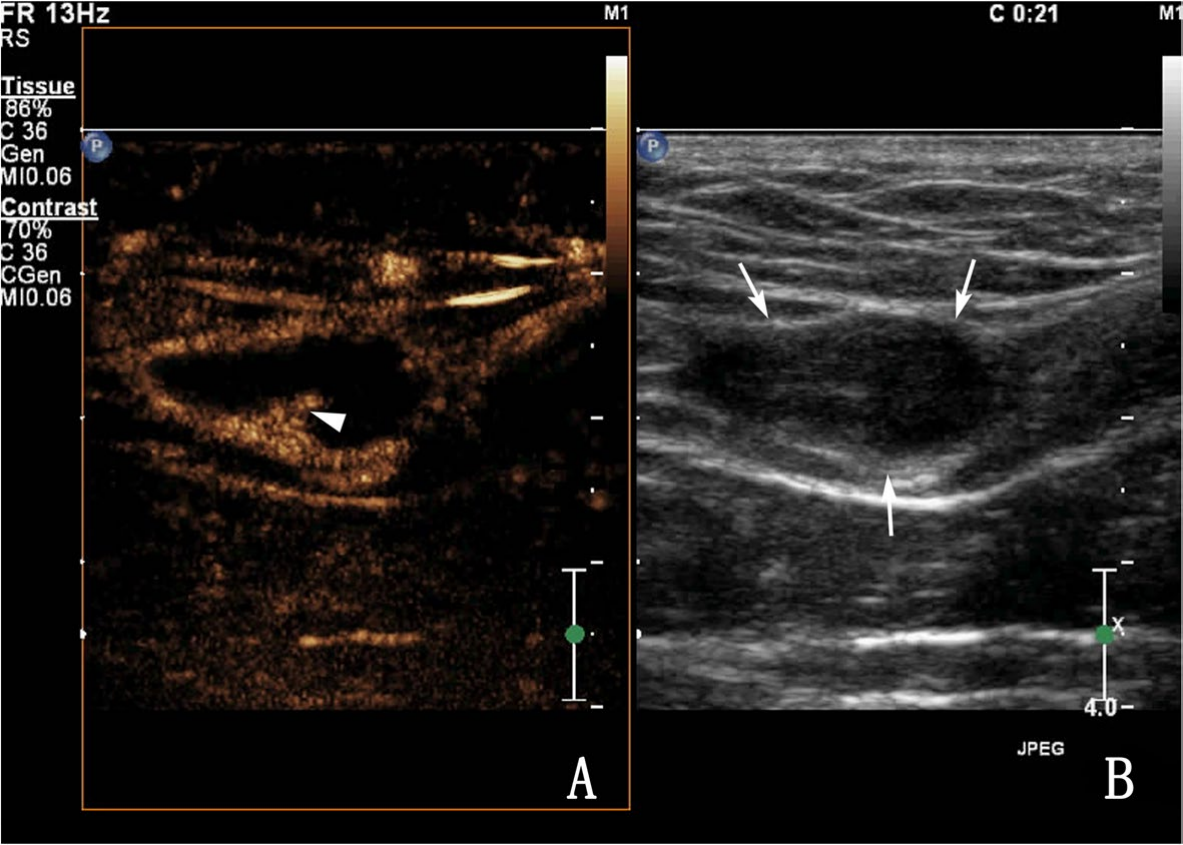
A 45-year-old male with tuberous vas deferens tuberculosis. **A** Routine ultrasonography showing a low-echo-type lesion (coarse arrow). **B** Contrast-enhanced ultrasonography showing heterogeneous enhancement with ring enhancement of a nodule (the small arrow shows a nodule-in-nodule) (Type IIc)

According to the pathological process and CEUS findings, tuberous VD TB was roughly divided into type I and type II, and type II was divided into three subtypes: Types IIa, IIb, and IIc. Among the patients with type I, CEUS showed homogeneous enhancement of the nodules in three patients who had the hypoechoic type by routine US, and the area after enhancement was similar to that observed by routine US (Fig. 2). Among the patients with type II, CEUS showed inhomogeneous enhanced nodules in 16 patients, including 9 with the hypoechoic type and 7 with the mixed echoic type by routine US. Among the 16 patients with type II, 11 were subclassified as type IIa (septal enhancement) in which CEUS showed inhomogeneous enhancement within the nodule, with septal enhancement, honeycomb, and petal-like enhancement (Fig. 3); 2 were subclassified as type IIb (circular enhancement) in which CEUS showed marginal enhancement of the nodule, with no internal enhancement zone; and four patients were subclassified as type IIc (nodule-in-nodule enhancement) in which CEUS showed inhomogeneous enhancement within the nodule, with only marginal and local enhancement within the nodule and no enhancement in most other parts within (Fig. 4).

The incidence rates of tuberous VD TB for the type I and type II nodule types with transverse diameters of the lesions are shown in Table 2.

**Table 2:** Proportion of type I and type II nodules with a transverse diameter of 1.0 cm as the boundary (%)

Size	Type I	Type II
> 1.0 cm	0	100% (11/11)
< 1.0 cm	37.5% (3/8)	62.5% (5/8)
Total	16.7% (3/19)	84.2% (16/19)

### Tuberous VD TB-positive diagnostic rates of CEUS and US

The positive diagnostic rate was 89.5% for CEUS and 68.4% for US, but the difference was not significant (χ2 = 2.533; *P* = 0.111).The tuberous VD TB-positive rates of CEUS and US are shown in Table 3.

**Table 3:** Positive rates of CEUS and US in tuberous vas deferens tuberculosis

Diagnostic result	Diagnostic methods		Total
	CEUS	US	
Positive (n)	17	13	30
Negative (n)	2	6	8
Total (n)	19	19	38
Positive diagnostic rate (%)	89.5	68.4	78.9

Contrast between CEUS and US, χ^2^ = 2.533, *P* = 0.111

## Discussion

Clinically, the most common tuberculosis of the male genital system is epididymal tuberculosis, whereas at autopsy, the most common type is prostate tuberculosis [8]. Given that the VD lies between the epididymis and prostate, the VD may be a factor in the relationship between prostatic and epididymal tuberculosis.

The incidence of VD TB in extrapulmonary tuberculosis is very low because it is a chronic process, so in most of the patients, a scrotal mass or scrotal pain, with a feeling of swelling in the perineum, are inadvertently found. Occasionally, patients have come to our hospitals because of a mass in the groin, and most of these patients had a history of tuberculosis or had typical tuberculosis symptoms. All patients in this study had a history of tuberculosis or were complicated with tuberculosis of other systems.

CEUS is a new diagnostic technology developed in recent years that can accurately determine the blood supply in lesions, and it is widely used in the US diagnosis of various viscera lesions [9–11]. Tuberous VD TB usually shows hypoechoic and mixed echoic types, and the mixed echoic images are usually consistent with the inhomogeneous enhanced images. In the present study, 9 of the 12 patients with hypoechoic tuberous VD TB on US showed inhomogeneous enhancement, and CEUS more clearly showed the tuberous VD TB pathological state. This finding may be related to the formation of caseous necrosis during the development of VD TB and to the thick necrotic material. The resulting ultrasonographic appearance is hypoechoic, which may also be because the nodules of caseous material within are scattered with a limited distribution, which makes them hard to distinguish by US.

In this study, 16 patients with tuberous VD TB showed inhomogeneous enhancement, 11 of which had transverse diameters > 1 cm. The proportion of inhomogeneous enhancement increased when the lesion’s transverse diameter was > 1 cm, whereas the transverse diameters of the nodules in the three patients with homogeneous enhancement of VD TB were all < 1 cm and observed in convalescing patients after they had undergone anti-tuberculosis treatment. We believe that the low incidence rate of VD TB with homogeneous enhancement is related to the chronic process of VD TB and small VD TB lesions in the early disease stage. Therefore, these small lesions are difficult to be felt by patients, and there are no obvious acute inflammatory manifestations, such as redness, swelling, heat, and pain. It is easy for some patients to be negligent, and most patients visit a doctor only after touching or noticing a groin mass. In the present study, all patients with uniformly enhanced VD TB were convalescing during the follow-up process, but this finding is insufficient to show that homogeneous enhancement is characteristic of patients with VD TB in the convalescent period. Studies with a larger patient sample would be needed to confirm this possibility, especially if they include patients with early VD TB [12]. The pathological process leading to nodules showing inhomogeneous enhancement of VD TB during disease progression is as follows: exudation, caseous necrosis, and thickening of the VD wall lead to formation of nodules that can expand to various sizes. In this study, the largest nodule was 4.3 × 2.8 cm. When the transverse diameter of the nodule was > 1 cm, the percentage of patients presenting with type II was 100%, and when the diameter of the nodule was < 1 cm, the percentage of type I was 37.5% (3/8) and of type II was 62.5% (5/8). However, the transverse diameter threshold of nodules for liquefied necrosis of VD TB remains to be confirmed in studies with a larger number of patients. CEUS showed inhomogeneous enhancement, suggesting internal necrosis, which was more likely to be found by CEUS than by routine US. Among the 19 nodular tuberculous vesicles, CEUS showed 16 nodules with inhomogeneous enhancement, whereas routine US showed 7 nodules with mixed echo.

Given the history of tuberculosis, compared with routine US, CEUS has a higher positive diagnostic rate for tuberous VD TB. The positive diagnostic rate of CEUS in the present study was 89.5%, but this result needs to be confirmed in multi-center studies. However, in the present study, there was no significant difference (*P* > 0.05) in the diagnostic value of CEUS and of routine US for tuberous VD TB, which may be because of the small number of patients in this study. Therefore, more multi-center data from a larger number of patients are needed to determine if there are significant differences between CEUS and routine US.

There is a discontinuous anechoic area in the VD distal to the nodule, indicating VD obstruction, and this phenomenon can be easily shown by US and CEUS. The size and number of nodules varied in different patients, and the size and number of bilateral nodules of VD TB in the same patient could also vary. In this study, the maximum number of nodules in one patient with VD TB was five. By CEUS, tuberous VD TB mostly showed inhomogeneous enhancement of nodules in 84.2% (16/19) of the patients in this study.To ensure the accuracy of CEUS images, interference from the spermatic vein must be avoided during imaging, and the spermatic vein should be accurately distinguished to avoid mistaking it for the thickened wall of the VD [2]. In this study, none of the tuberous VD TB had broken through the spermatic capsule and extended outward to form a sinus tract or peripheral abscess, but the tuberous VD TB was closely related to various structures in the spermatic cord and had poor mobility. The tuberous portion felt hard when probed, and it was not easy to be deformed or pushed.

## Conclusion

From what has been discussed above non-homogeneous enhancement was most often observed in CEUS of tuberous VD TB, of which type IIa was the most common. We believe that septal enhancement of tuberous VD TB is characteristic of CEUS imaging, which deserves attention in future examinations. Compared with routine US, CEUS was more likely to show internal necrosis of nodules. When the nodule diameter of tuberous VD TB was > 1 cm, CEUS showed inhomogeneous enhancement, indicating necrosis inside the nodule, which is helpful for the diagnosis of tuberous VD TB.

## Data Availability

All data generated or analyzed during this study are included in this published article.
